# Sub-macroscopic skin presentation of acromegaly and effect of pituitary tumor surgery: A study using dermatoscopy and ultra-high-frequency ultrasound

**DOI:** 10.3389/fendo.2022.1093942

**Published:** 2023-01-10

**Authors:** Xiaopeng Guo, Yukun Wang, Yong Yao, Xinjie Bao, Lian Duan, Huijuan Zhu, Bing Xing, Jie Liu

**Affiliations:** ^1^ Department of Neurosurgery, Center for Pituitary Surgery, China Pituitary Disease Registry Center, China Pituitary Adenoma Specialist Council, Peking Union Medical College Hospital, Chinese Academy of Medical Sciences and Peking Union Medical College, Beijing, China; ^2^ Department of Dermatology, National Clinical Research Center for Dermatologic and Immunologic Diseases, State Key Laboratory of Complex Severe and Rare Diseases, Peking Union Medical College Hospital, Chinese Academy of Medical Science and Peking Union Medical College, Beijing, China; ^3^ Department of Endocrinology, Key Laboratory of Endocrinology of the Ministry of Health, Peking Union Medical College Hospital, Chinese Academy of Medical Sciences and Peking Union Medical College, Beijing, China

**Keywords:** acromegaly, dermatoscopy, ultra-high-frequency ultrasound, skin, surgery

## Abstract

**Objective:**

Excessive growth hormone and insulin-like growth factor 1 contribute to cutaneous changes in acromegaly. We investigated the sub-macroscopic skin manifestation of acromegaly patients and explored its reversibility upon hormone reduction after pituitary adenoma surgery.

**Design:**

Prospectively cohort study.

**Methods:**

We enrolled 26 patients with acromegaly and 26 patients with non-functioning pituitary adenomas undergoing pituitary adenomectomy at Peking Union Medical College Hospital from July 2021 to March 2022. Skin presentations were evaluated by dermatoscopy and ultra-high-frequency ultrasound before and after surgery.

**Results:**

Skin thickening, follicular plugs, perifollicular pigmentations, perifollicular orange haloes, red structureless areas, increased hair shafts, honeycomb-like pigmentations, widened dermatoglyphics, dilated appendage openings, excessive seborrhea, hyperhidrosis, enlarged pores, and acne-like lesions were commonly occurring in acromegaly patients, and their incidences were higher than the controls (P<0.05). At 3-month follow-up after surgery, the thickness of skin reduced (4.0 ± 0.4 to 3.7 ± 0.4, P=0.007), the incidences of hyperhidrosis (92.3% to 69.2%, P=0.035) and acne-like lesions (53.8% to 26.9%, P=0.048) declined, and the severity of multiple cutaneous lesions improved. Patients with surgical endocrine remission (53.8%) had greater declines in the thickness of skin than those without remission. Patients with improvement of >1 skin lesions were younger (P=0.028) and had higher baseline GH levels (P=0.021) than those with improvement of ≤1 skin lesion.

**Conclusions:**

Dermatoscopy and ultra-high-frequency ultrasound provided augmented visual examination of the cutaneous changes in acromegaly. Some of the skin lesions could improve or reverse after pituitary surgery. Baseline GH levels, age, and endocrine remission were correlated with skin improvement at 3-month follow-up.

## 1 Introduction

Pituitary tumor accounts for 17.2% of primary brain and other central nervous system neoplasms and is the second most common tumor type (1). Pituitary somatotroph adenoma produces and releases excessive growth hormone (GH) into the circulation and consequently leads to a high level of serum insulin-like growth factor 1 (IGF-1) (2). Continuous stimulation from the hypersecretion of GH and IGF-1 results in acromegaly with a series of clinical presentations, including facial appearance changes, extremity enlargement, bone modifications, and systemic complications (3–8). Skin is a well-organized endocrine organ expressing GH and IGF-1 receptors (9). Hormone (GH/IGF-1) excess in acromegaly leads to various skin manifestations including skin coarsening, skin puffiness, skin thickening, acne, skin tag, hypertrichosis, hyperidrosis, and oily skin (5, 10). In clinical practice, the diagnosis of acromegaly was mainly initiated by above skin presentations. After treatment, some skin lesions are reversible by naked-eye inspection upon the decrease of serum GH/IGF-1 levels, while some lesions are not (5, 6, 9, 11).

Compared with unaided eyes, dermatoscopy provides a sub-macroscopic view on both pigmented and non-pigmented skin lesions and is considered the state-of-the-art non-invasive diagnostic method with high accuracy and wide availability (12–14).Studies have shown that dermatoscopy increases the diagnostic sensitivity for melanoma in comparison with inspection by unaided eyes (15).Additionally, ultra-high-frequency ultrasound (UHFUS) with a transducer frequency of 50 MHz or higher produces high-resolution observations of tissues closer to the skin surface (16–19). Although efforts have been made to satisfy early detection of acromegaly, its diagnosis has still been delayed resulting in increased morbidity and mortality (20). We proposed that dermatoscopy and UHFUS allowed sub-macroscopic visualization on higher magnification to pick up subtle changes that were not visible to naked eyes and thus had great potential to aid in early detection and response assessment to surgical treatments for acromegaly.

Little is known about the sub-macroscopic skin manifestations of acromegaly patients and whether these skin lesions are reversible after pituitary tumor surgery. In this prospective cohort study, we enrolled patients with active acromegaly and age- and sex-matched patients with non-functioning pituitary adenomas (NFPAs) to address the following issues: 1) what are the sub-macroscopic presentations of acromegaly-related cutaneous changes; 2) whether these skin lesions are reversible after pituitary adenomectomy; and 3) are there clinical correlations with greater skin improvements after surgery.

## 2 Subjects and methods

### 2.1 Study population

Adult acromegaly patients of either gender who were admitted to Peking Union Medical College Hospital Neurosurgery Department for pituitary adenoma resection from July 1, 2021, to March 31, 2022, were prospectively enrolled. Diagnosis of acromegaly was based on the Endocrine Society Clinical Practice Guideline (21): IGF-1 exceeding the age- and sex-matched upper limit of normal (ULN), and inability to suppress GH to 1.0 ng/ml after oral glucose tolerance test (OGTT). During the same period, age- and sex-matched patients with NFPAs who were scheduled pituitary surgery were included as controls. Both treatment-naïve and recurrent patients were enrolled. The exclusion criteria were as follows: 1) patients with other diseases involving the skin; 2) patients with a history of any treatments that might potentially influence the skin; and 3) patients who refused to participate in this study.

### 2.2 Ethics

This study was conducted following the tenets of the Helsinki declaration and approved by the Institutional Review Board at Peking Union Medical College Hospital (NO. JS-1233). All patients signed written informed consent before enrollment.

### 2.3 Study design

This was a prospective, longitudinal cohort study. Clinical data including age at diagnosis, sex, body mass index, disease duration, hormone levels, function of each hypothalamus-pituitary-organ axis, radiological tumor features on magnetic resonance imaging (MRI), and tumor Ki-67 index were recorded. Disease duration was calculated from the onset of acromegaly-related symptoms to the clinical diagnosis. Macroadenomas were defined as tumors with a maximal diameter over 1cm. Cavernous sinus invasion was recognized if the maximal Knosp grade of either side of tumor on the coronal MRI was >2 (22). IGF-1%ULN was transformed from the absolute value using age-sex matched ULNs at our institute (7) to standardize IGF-1 comparisons between patients.

Patients of both groups underwent pituitary tumor resection after exclusion of surgical contraindications, and all the tumors were confirmed by the histology and immunohistochemical staining. Postoperative follow-up at three months was scheduled for all acromegaly patients. Endocrine remission of acromegaly was recognized if fasting GH <1.0 ng/ml or GH nadir <0.4 ng/ml after OGTT, and normalized IGF-1 (23).

### 2.4 Macroscopic and sub-macroscopic skin assessment

**Figure 1:**
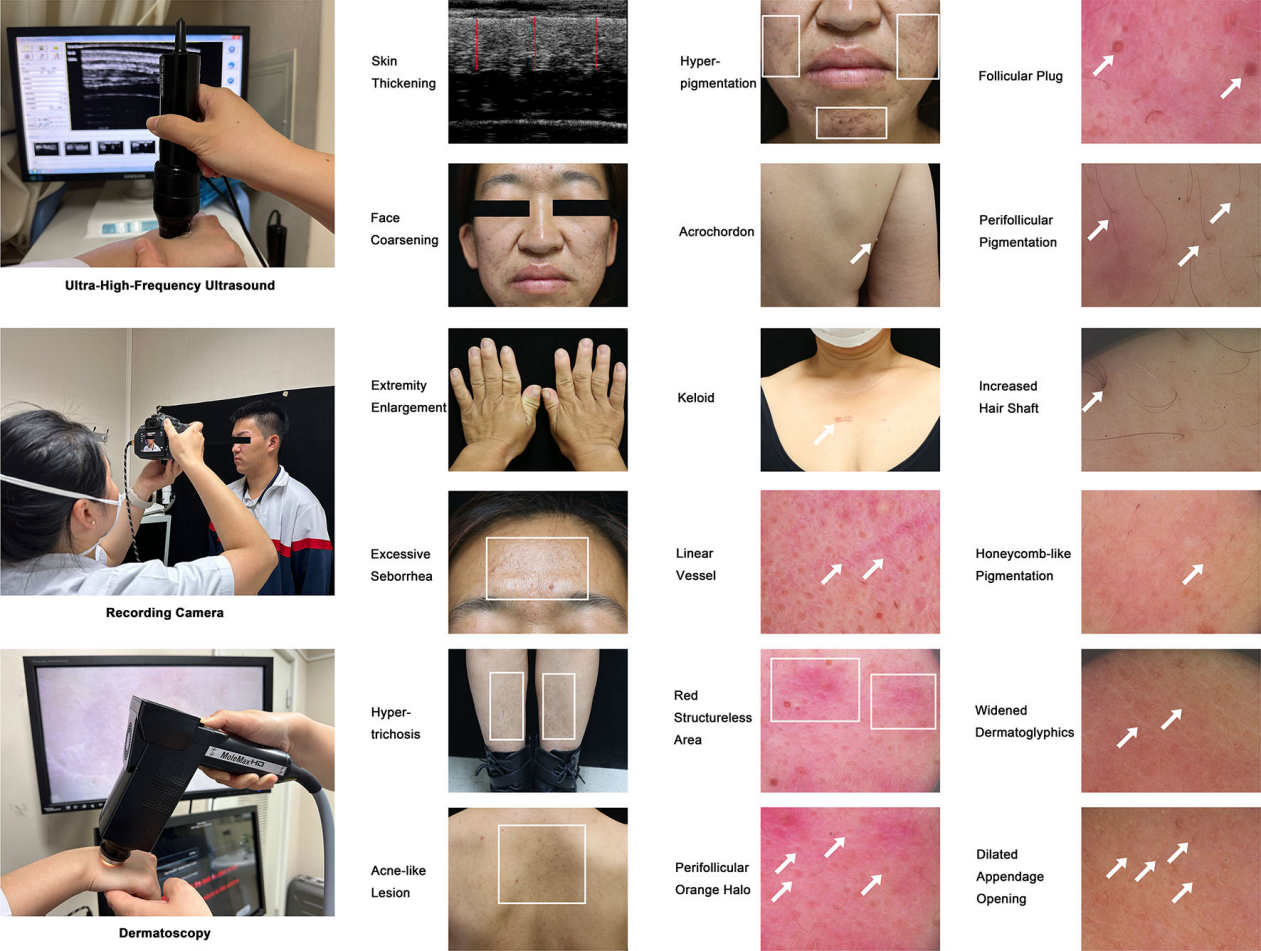
Ultrasonic, Megascopic, and Dermatoscopic Skin Presentations of Acromegaly. This figure shows the devices including UHFUS, camera, and dermatoscopy utilized to assess acromegaly-related cutaneous manifestations in this study and contains 18 representative images showing the sub- and macroscopic skin lesions of acromegaly patients. The illustrated cutaneous manifestations include thickened skin (compared with the controls using the UHFUS), face coarsening, extremity enlargement, excessive seborrhea, hypertrichosis, acne-like lesion (on the upper back), hyper-pigmentation (on the face), acrochordon, keloid, and sub-macroscopic skin lesions shown by dermatoscopy (linear vessel, red structure area, perifollicular orange halo, follicular plug, perifollicular pigmentation, increased hair shaft, honeycomb-like pigmentation, widened dermatoglyphics, and dilated appendage opening). Arrows and rectangles were used to highlight the typical skin lesions.

Skin presentations were recorded using camera, UHFUS, and dermatoscopy for both groups before surgery and for acromegaly patients at 3-month follow-up (**Figure 1**). Naked-eye inspection of skin presentations included face coarsening, extremity enlargement, thickened skin, excessive seborrhea, hypertrichosis, enlarged pores, acne-like lesion, hyper-pigmentation, facial erythema, acrochordon, and keloid.

Sub-macroscopic skin presentations included the thickness of skin assessed by a UHFUS device (50mHz, Tianjin Media Medical Technology Co., Ltd, Tianjin, China), and linear vessel, red structureless area, perifollicular orange halo, follicular plug, perifollicular pigmentation, increased hair shaft, honeycomb-like pigmentation, widened dermatoglyphics, and dilated appendage opening assessed by a dermatoscopy device (MoleMax HD version 1.0, Digital Image Systems, Vienna, Austria). The thickness of the skin was assessed in the middle of the patient’s upper back and was calculated using the average value of three perpendicular lines drawn from the surface to the dermal-subcutaneous tissue junction. The skin presentations by dermatoscopy were captured on the face, chest, back, limbs, hands, and feet.

Each skin lesion was stored in the database and further evaluated independently by two dermatologists with more than 5-year clinical experience who were blind to patient clinical information. All lesions were quantitatively marked with a specific degree: 0-none, 1-mild, 2-medium, or 3-severe. Improvement of skin lesions was identified if its degree changed from 3-severe to 2-moderate/1-mild/0-none, from 2-moderate to 1-mild/0-none, or from 1-mild to 0-none. Among all the skin lesions that were evaluated in one patient, if two or more lesions were improved after surgery, we defined that this patient had skin improvement.

### 2.5 Statistical analyses

Categorical variables were shown as numbers and percentages, and their comparisons were performed using the chi-squared test. Continuous variables were presented as the means ± standard deviations or medians plus interquartile range (25th and 75th percentile) according to data distribution. Student’s t-test was used to assess the differences between normally distributed continuous variables, and Mann-Whitney U test was used with variables that failed the normality test. P<0.05 indicated statistical significance. SPSS Statistics (version 26.0, IBM, USA) was used to analyze the data. Prism (version 9.3.1, GraphPad, USA) was used to generate graphs.

## 3 Result

### 3.1 Study participants

A total of 26 acromegaly patients and 26 patients with NFPAs were enrolled. Mean age was 47.5 ± 11.7 years of acromegaly patients (female, 17 of 26 [65.4%]) and 44.0 ± 12.6 years of the controls (female, 19 of 26 [73.1%]). The diagnosis of acromegaly was delayed with a longer disease duration compared with patients with NFPAs (median, 54 vs. 6, months, P<0.001). The maximal tumor diameter was shorter in acromegaly patients than that in the controls (17.0 ± 6.2 vs. 21.1 ± 6.3, mm, P=0.022). Before surgery, the median GH level was 17.3 (8.1, 36.9) ng/ml, GH nadir level was 10.0 (5.3, 20.3) ng/ml, and IGF-1%ULN was 2.1 (1.7, 2.8). The incidence of hyperprolactinemia, hypothyroidism, hypogonadism, and adrenal insufficiency were similar between groups (**Table 1**). All acromegaly patients completed the 3-month follow-up, and 14 patients (53.8%) satisfied the criteria of endocrine remission.

**Table 1:** Clinical characteristics of acromegaly patients and the controls.

	Acromegaly Patients (n=26)	Controlled Patients (n=26)	P
Baseline Characteristics			
Age at diagnosis, years	47.5 ± 11.7	44.0 ± 12.6	0.314
Male, n (%)	9 (34.6)	7 (26.9)	0.548
Body mass index, kg/m^2^	25.6 ± 3.3	25.6 ± 3.6	0.947
Disease duration, months	54 (48, 88)	6 (2, 24)	<0.001
Tumor maximal diameter, cm	17.0 ± 6.2	21.1 ± 6.3	0.022
Macroadenoma, n (%)	24 (92.3)	26 (100)	0.471
Maximal Knosp grade, n	2 (1, 3)	2 (1, 3)	1.000
Cavernous sinus invasion, n (%)	8 (30.8)	9 (34.6)	0.768
Ki-67 index of the tumor, %	2 (1, 3)	3 (1, 4)	0.203
Recurrent tumor, n (%)	6 (23.1)	2 (7.7)	0.249
GH level, ng/ml	17.3 (8.1, 36.9)	0.1 (0.1, 0.4)	<0.001
GH nadir after OGTT, ng/ml	10.0 (5.3, 20.3)	–	–
IGF-1 level, ng/ml	552.0 (474.5, 704.3)	142.5 (110.5, 174.0)	<0.001
IGF-1%ULN, %	2.1 (1.7, 2.8)	0.5 (0.4, 0.6)	<0.001
Hyperprolactinemia, n (%)	3 (11.5)	5 (19.2)	0.701
Central hypothyroidism, n (%)	4 (15.4)	0 (0)	0.118
Central hypogonadism, n (%)	4 (15.4)	3 (11.5)	1.000
Central adrenal insufficiency, n (%)	0 (0)	1 (3.8)	1.000
Assessment at 3-month follow-up			
Endocrine remission, n (%)	14 (53.8)	–	–
GH level, ng/ml	1.0 (0.7, 3.7)	–	–
GH nadir after OGTT, ng/ml	0.4 (0.3, 1.7)	–	–
IGF-1 level, ng/ml	255.0 (221.0, 326.0)	–	–
IGF-1% ULN, %	1.0 (0.9, 1.3)	–	–

Continuous variables are presented as means ± standard deviations if normally distributed or medians (25^th^ and 75^th^ quartile) if not normally distributed.

GH, growth hormone; IGF-1, insulin-like growth factor 1; OGTT, oral glucose tolerance test; ULN, upper limit of normal.

### 3.2 Skin presentations of acromegaly

Representative macroscopic and sub-macroscopic skin presentations of acromegaly were presented in **Figure 1**. From a macroscopic view, more acromegaly patients had thickened skin, face coarsening, enlarged hands and feet, hypertrichosis, enlarged pores, excessive seborrhea, hyperhidrosis, hyperpigmentation, and acne-like lesions compared with the patients with NFPAs (all P ≤ 0.01, **Table 2**). Under the dermatoscopy, more acromegaly patients had red structureless area, perifollicular orange halo, follicular plug, perifollicular pigmentation, increased hair shafts, honeycomb-like pigmentation, widened dermatoglyphics, and dilated appendage opening than the controls (all P<0.01, **Table 2**). Using the UHFUS, mean skin thickness on the upper back was 4.0 ± 0.4 mm in acromegaly patients and 3.5 ± 0.5 mm in the controls (P<0.001, **Table 2**).

**Table 2:** Megascopic, dermatoscopic, and ultrasonic skin presentations of acromegaly patients and the effect of transsphenoidal pituitary tumor surgery.

	Controlled patients (n=26)	Acromegaly Patients (n=26)			
		Baseline	P _baseline vs. controls_	Post-operation	P _postop. vs. baseline_
Megascopic Skin Presentations					
Thickened skin, n (%)	0 (0)	26 (100) ↑	<0.001	26 (100)	1.000
Face coarsening, n (%)	0 (0)	25 (96.2) ↑	<0.001	25 (96.2)	1.000
Enlarged hands and feet, n (%)	0 (0)	26 (100) ↑	<0.001	26 (100)	1.000
Enlarged pores, n (%)	2 (7.7)	23 (88.5) ↑	<0.001	23 (88.5)	1.000
Hypertrichosis, n (%)	1 (3.8)	11 (42.3) ↑	0.001	11 (42.3)	1.000
Acrochordon, n (%)	0 (0)	4 (15.4) ↑	0.118	4 (15.4)	1.000
Keloid, n (%)	0 (0)	5 (19.2) ↑	0.060	5 (19.2)	1.000
Excessive seborrhea, n (%)	5 (19.2)	25 (96.2) ↑	<0.001	20 (76.9) ↓	0.104
Hyperhidrosis, n (%)	1 (3.8)	24 (92.3) ↑	<0.001	18 (69.2) ↓	0.035
Acne-like lesion, n (%)	5 (19.2)	14 (53.8) ↑	0.010	7 (26.9) ↓	0.048
Hyperpigmentation, n (%)	2 (7.7)	18 (69.2) ↑	<0.001	17 (65.4) ↓	0.768
Facial erythema, n (%)	16 (61.5)	21 (80.8) ↑	0.126	16 (61.5) ↓	0.126
Dermatoscopic Skin Presentations					
Linear vessel, n (%)	9 (34.6)	10 (38.5) ↑	0.575	8 (30.8) ↓	0.560
Red structureless area, n (%)	17 (65.4)	26 (100) ↑	0.003	24 (92.3) ↓	0.471
Perifollicular orange halo, n (%)	7 (26.9)	23 (88.5) ↑	<0.001	19 (73.1) ↓	0.159
Follicular plug, n (%)	1 (3.8)	19 (73.1) ↑	<0.001	17 (65.4) ↓	0.548
Dilated appendage opening, n (%)	2 (7.7)	18 (69.2) ↑	<0.001	17 (65.4) ↓	0.768
Perifollicular pigmentation, n (%)	6 (23.1)	24 (92.3) ↑	<0.001	24 (92.3)	1.000
Honeycomb-like pigmentation, n (%)	10 (38.5)	25 (96.2) ↑	<0.001	25 (96.2)	1.000
Increased hair shafts, n (%)	1 (3.8)	14 (53.8) ↑	<0.001	14 (53.8)	1.000
Widened dermatoglyphics, n (%)	1 (3.8)	21 (80.8) ↑	<0.001	21 (80.8)	1.000
Ultrasonic Skin Presentation					
Skin thickness, mm	3.5 ± 0.5	4.0 ± 0.4 ↑	<0.001	3.7 ± 0.4 ↓	0.007

↑/↓ indicated the trend of the rate changes of skin presentations or skin thickness between groups.

P<0.05 indicated the between-group difference was statistically significant.

### 3.3 Changes in rates of skin lesions and skin thickness after surgery

At 3-month follow-up after surgery, the percentages of acromegaly patients who had excessive seborrhea, hyperhidrosis, acne-like lesion, hyperpigmentation, facial erythema, linear vessel, red structureless area, perifollicular orange halo, follicular plug, and dilated appendage openings had a trend of decline (**Table 2**). Among these lesions, the decline of hyperhidrosis (92.3% to 69.2%, P=0.035) and acne-like lesion (53.8% to 26.9%, P=0.048) were significant. The postoperative skin thickness decreased remarkably (4.0 ± 0.4 to 3.7 ± 0.4, P=0.007) to the baseline level (3.7 ± 0.4 vs. 3.5 ± 0.5, P=0.207).

### 3.4 Effect of endocrine remission on the remission of skin lesions

The postoperative levels of GH (median, 17.3 vs. 1.0, ng/ml, P<0.001), GH nadir (median, 10.0 vs. 0.4, ng/ml, P<0.001), and IGF-1%ULN (median, 2.1 vs. 1.0, P<0.001) were all significantly decreased than the baseline. Postoperative median levels of GH (0.7 vs. 3.9, ng/ml, P<0.001), GH nadir (0.3 vs. 1.8, ng/ml, P<0.001), and IGF-1%ULN (0.9 vs. 1.4, P<0.001) were lower in patients in endocrine remission than those not in remission. For all patients in both subgroups, the percentages of several skin lesions showed the trend of decline but were not significant (**Supplementary Table 1**). Skin thickness was significantly decreased in patients in remission (4.0 ± 0.4 to 3.6 ± 0.4, P=0.045) but was not significantly decreased in those not in remission (4.1 ± 0.4 vs. 3.8 ± 0.4, P=0.080).

### 3.5 Improvement of skin lesions after pituitary surgery

**Figure 2:**
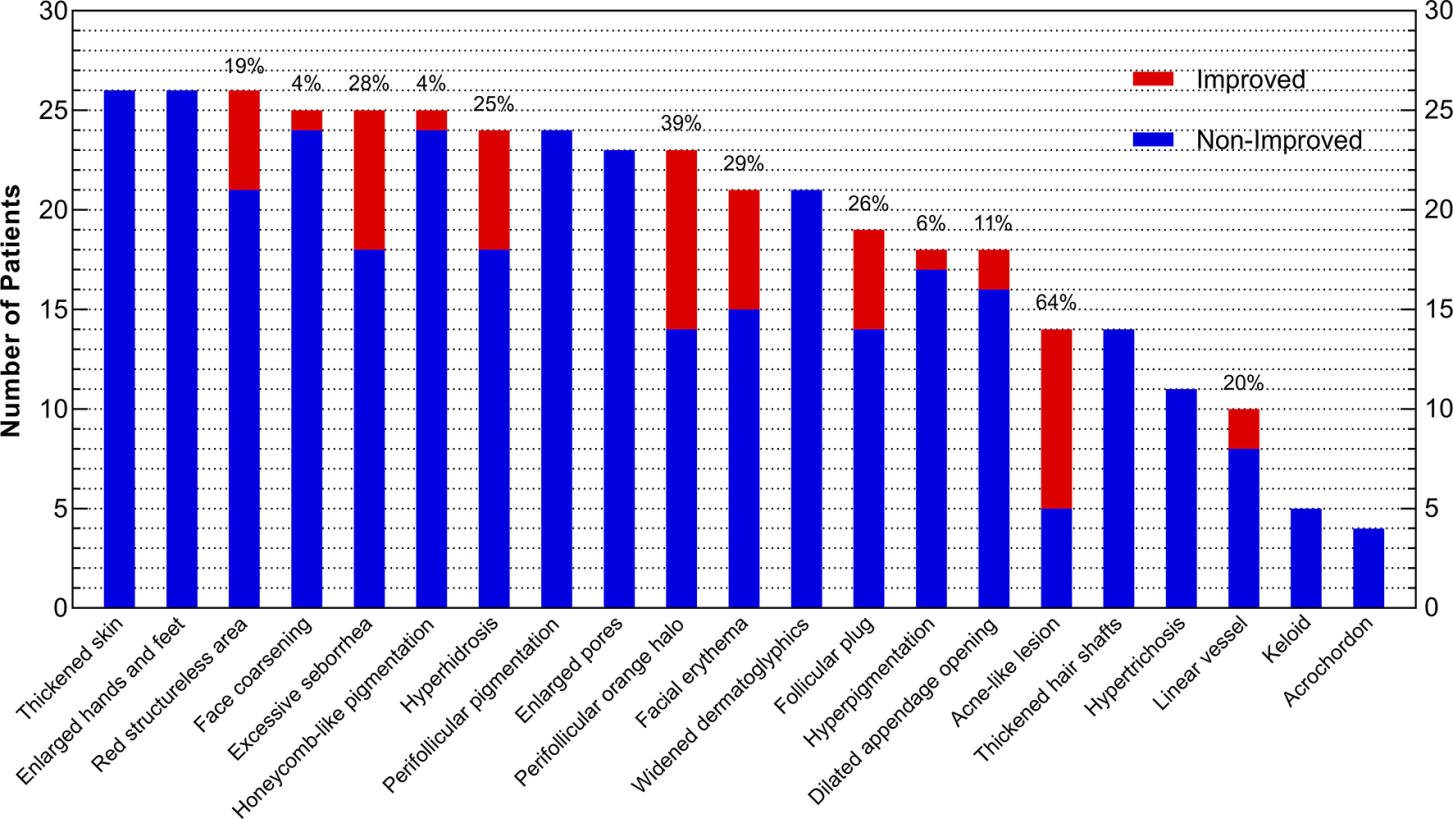
Improvement of Skin Lesions in Acromegaly Patients after Surgery. The x-axis presents 21 skin lesions, and the y-axis indicates the number of patients who had improvement (red) or non-improvement (blue) of skin lesions after surgery. The whole column (red plus blue) represents the number of patients with skin lesions before surgery, and the percentages laid on the top of columns mean the rates of skin lesion improvement (red) after surgery.

Acne-like lesion (64%), perifollicular orange halo (39%), facial erythema (29%), excessive seborrhea (28%), and follicular plug (26%) had the highest improvement rates among all skin lesions in acromegaly (**Figure 2**). Patients with improvements of 2 or more skin lesions were younger (42.5 ± 11.8 vs. 52.4 ± 9.6, years, P=0.028) and had a high baseline GH level (median, 36.3 vs. 8.8, ng/ml, P=0.021) than those with improvements of ≤1 skin lesion (**Table 3**).

**Table 3:** Clinical relevance of postoperative skin lesion improvement (>1/≤1) in acromegaly.

	Improvement>1 (n=13)	Improvement ≤ 1 (n=13)	P values
Age at diagnosis, years	42.5 ± 11.8	52.4 ± 9.6	0.028
Male, n (%)	6 (46.2)	3 (23.1)	0.410
Body mass index, kg/m^2^	26.0 ± 4.1	25.2 ± 2.5	0.578
Disease duration, months	48 (24, 88)	60 (48, 60)	0.609
Tumor maximal diameter, cm	18.3 ± 6.6	15.6 ± 5.7	0.276
Macroadenoma, n (%)	12 (92.3)	12 (92.3)	1.000
Maximal Knosp grade, n	2 (1, 3)	2 (2, 3)	0.582
Cavernous sinus invasion, n (%)	4 (30.8)	4 (30.8)	1.000
Ki-67 index of the tumor, %	3 (2, 3)	2 (1, 3)	0.893
Recurrent tumor, n (%)	4 (30.8)	2 (15.4)	0.642
Baseline GH level, ng/ml	36.3 (20.3, 51.6)	8.8 (5.8, 16.1)	0.021
Baseline GH nadir after OGTT, ng/ml	18.7 (9.3, 32.4)	5.8 (3.5, 11.0)	0.105
Baseline IGF-1 level, ng/ml	646 (552, 714)	482 (414, 548)	0.143
Baseline IGF-1% ULN, %	2.4 (1.9, 2.9)	2.0 (1.5, 2.3)	0.670
Hyperprolactinemia, n (%)	0 (0)	3 (23.1)	0.220
Central hypothyroidism, n (%)	2 (15.4)	2 (15.4)	1.000
Central hypogonadism, n (%)	2 (15.4)	2 (15.4)	1.000
Central adrenal insufficiency, n (%)	0 (0)	0 (0)	1.000
Endocrine remission, n (%)	7 (53.8)	7 (53.8)	1.000
Postoperative GH level, ng/ml	0.9 (0.8, 3.1)	1.0 (0.7, 3.9)	0.797
Postoperative GH nadir after OGTT, ng/ml	0.4 (0.3, 1.7)	0.4 (0.2, 1.5)	0.473
Postoperative IGF-1 level, ng/ml	283 (239, 387)	228 (203, 321)	0.084
Postoperative IGF-1% ULN, %	1.0 (0.9, 1.8)	1.0 (0.9, 1.2)	0.218

GH, growth hormone; IGF-1, insulin-like growth factor 1; OGTT, oral glucose tolerance test; ULN, upper limit of normal.

### 3.6 Clinical correlations of postoperative improvement of skin lesions

**Figure 3:**
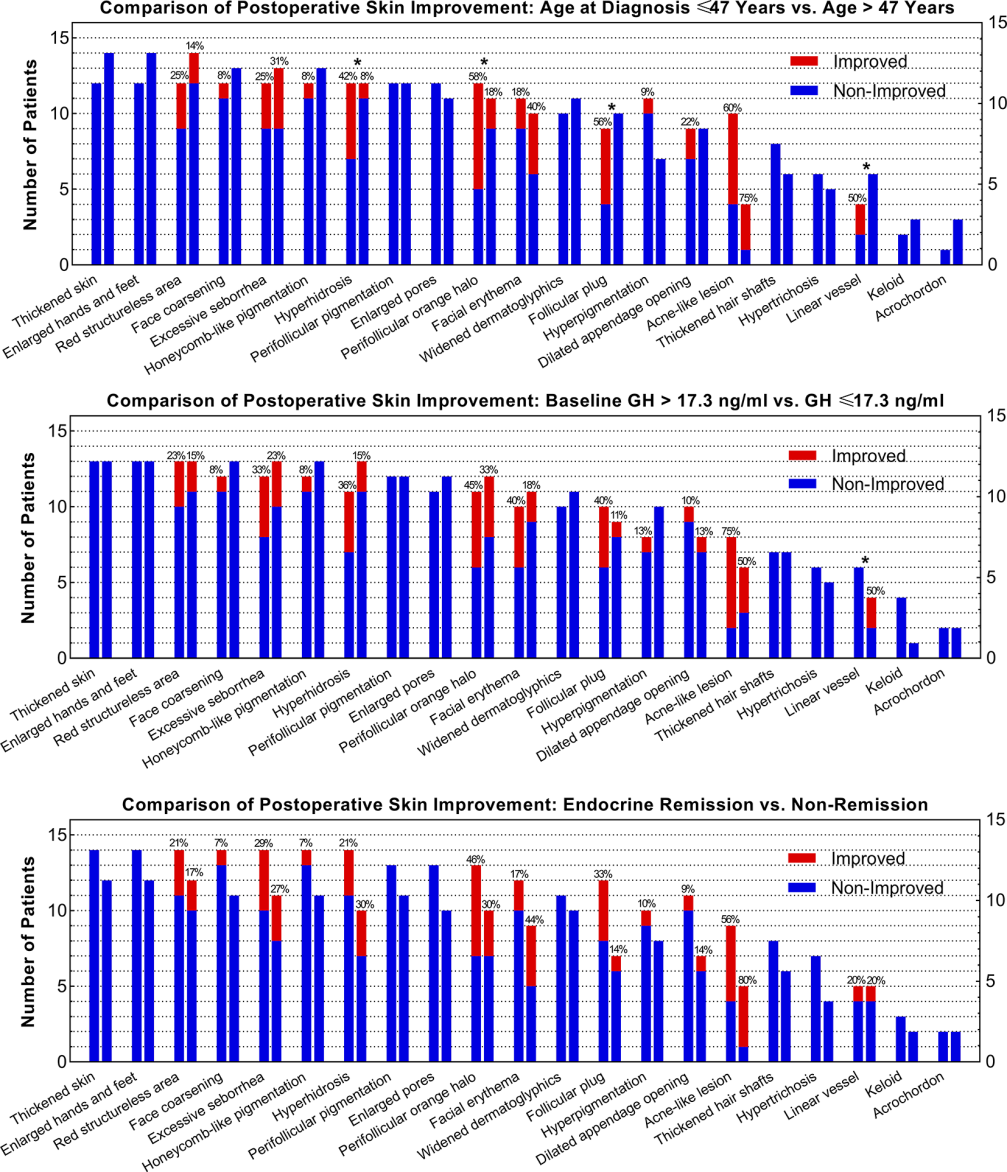
Comparisons of the Improvement Rate of Each Skin Lesion between Subgroups as Stratified by Age, Baseline GH Level, and Remission Status. ^*^ Indicates that the difference of the improvement rates of skin lesions between subgroups is over 30%.

Since age and baseline GH level was correlated with skin lesion improvement after surgery, we evaluated the improvement rate differences of all skin lesions in subgroups (**Figure 3**). Forty-seven years old for age and 17.3 ng/ml for preoperative GH levels were selected as median cut-off values. Patients aged ≤47 years had higher improvement rates of hyperhidrosis (42% vs. 8%), perifollicular orange halo (58% vs. 18%), follicular plug (56% vs. 0%), and linear vessel (50% vs. 0%) than those aged >47 years. Patients with a baseline GH ≤17.3 ng/ml had a higher rate of linear vessel improvement (50% vs. 0%) than those with a baseline GH >17.3 ng/ml.

## 4 Discussion

Dermatoscopy and UHFUS provide augmented visual examination of subtle cutaneous changes and set the foundation for early detection of the diseases of which skin presentations can imply important diagnostic clues (12, 24, 25). To our knowledge, the current study is the first to investigate the cutaneous presentations in acromegaly patients using dermatoscopy and UHFUS and explore the efficacy of hormone restoration on sub-macroscopic skin lesions after pituitary surgery. The results of this study provided evidence of the chronic effects of excessive GH and IGF-1 on human skin under a magnifying view and implied the reversibility of acromegaly-related skin presentations upon withdrawal of GH/IGF-1 hypersecretion.

Cells and appendant organs of the skin, including epidermal and follicular keratinocytes, papilla cells, fibroblasts, endothelial cells, melanocytes, sebocytes, and sweat glands, are all influenced by hormones (9). Naked-eye inspections of the cutaneous presentations of acromegaly include skin thickening, skin edema, skin wrinkling, face coarsening, neoplastic tags, excessive seborrhea, hyperhidrosis, and pigmentation (5, 10, 26–29). In this study, extremity enlargement, facial feature changes, hyperpigmentation, oily skin, and hyperhidrosis were detected in the majority of acromegaly patients while hypertrichosis and skin tags were detected in a small proportion of patients, in accordance with previous studies (9, 29–31).

Dermatoscopy and UHFUS are easy to use and are both non-invasive devices that enable the observation of subtle skin changes. In this study, red structureless area was seen on the faces of all patients. Honeycomb-like pigmentation, perifollicular pigmentation, perifollicular orange halo, and widened dermatoglyphics were noted in over 80% of the patients. Follicular plugs, increased hair shafts, and dilated appendage openings were detected in more than half of the patients. The incidences of all above skin lesions were higher compared to the controlled patients who were not influenced by the hypersecretion of GH and IGF-1. The skin of acromegaly patients is also remarkably thickened detected by UHFUS. These augmented visual examinations of skin lesions may have the potential for early detection of acromegaly before the emergence of obvious presentations by naked eyes like were used in the diagnosis of melanoma (12, 14, 24).

Upon reduction of serum levels of GH and IGF-1 after treatment, according to previous publications, reversibility of acromegaly-related skin lesions assessed by unaided eyes varied (5, 6, 9, 11). Apart from the naked-eye inspection, dermatoscopy and UHFUS were recently utilized in the follow-up of post-treatment sub-macroscopic skin responses (19, 32). From our study, skin thickness and the incidences of hyperhidrosis and acne-like lesions were significantly decreased, while the incidences of several other skin lesions decreased but were not significant and the other lesions remained the same with the baseline. One explanation for this is that the process of skin remodeling may undergo a longer duration over 3 months since the formation of acromegaly-related skin presentations experiences a chronic course for several years before the diagnosis of the disease (20, 33).

Endocrine remission as shown in our previous nationwide investigation was correlated with greater improvement in facial feature changes, hyperhidrosis, and hairness after a 5-year follow-up (33). Our results from this study revealed that endocrine remission contributed to a greater reduction in the thickness of skin of acromegaly patients, highlighting the rapid reversibility of the structures of the epidermis and dermis. However, the incidences of other skin lesions did not experience additional declines upon endocrine remission during 3 months.

Although most skin lesions were not completely remitted, the degree of severity of some lesions improved. We found that acne-like lesions, perifollicular orange halo, facial erythema, excessive seborrhea, and follicular plug improved at the 3-month follow-up in more than one-fifth of patients. We hypothesized that the subtle skin improvements evaluated by dermatoscopy and UHFUS might imply clinical skin improvement by unaided eyes years after treatment, which needed further validation in the prolonged follow-up.

Identifying clinical parameters that were correlated with skin lesion improvement could assist in patient consulting and postoperative skin care. We found that patients at a younger age or with higher baseline GH levels were more likely to experience better improvement of skin lesions. Therefore, older patients and those with a relatively lower baseline GH level might experience unsatisfactory skin lesion improvement and need additional medical intervention to accelerate the process of postoperative skin recovery.

This study has limitations. The sample size was small, leading to possible false negative results on skin lesion improvement after pituitary tumor surgery for acromegaly. Further studies with more participants are warranted to validate our results. The follow-up time was only three months, and a prolonged postoperative observation was required to acknowledge the reversibility of acromegaly-related skin presentation in the long run.

## 5 Conclusions

Dermatoscopy and UHFUS allowed visualization on higher magnification of the subtle, sub-macroscopic cutaneous presentations of acromegaly patients and skin responses to surgical treatment. Sub-macroscopic skin lesions were commonly occurring in acromegaly patients. Some lesions were completely reversed and some were improved at 3-month follow-up after pituitary surgery. Higher baseline GH levels, younger age at diagnosis, and endocrine remission were correlated with postoperative improvement in acromegaly-related skin lesions.

## Data availability statement

The original contributions presented in the study are included in the article/**Supplementary Material**. Further inquiries can be directed to the corresponding authors.

## Ethics statement

The studies involving human participants were reviewed and approved by Institutional Review Board at Peking Union Medical College Hospital. The patients/participants provided their written informed consent to participate in this study. Written informed consent was obtained from the individuals for the publication of any potentially identifiable images or data included in this article.

## Author contributions

XG, YW, BX, and JL designed the study. YW and JL performed the skin assessment and recorded data. YY, XB, LD, HZ, BX and JL monitored the entire procedure. XG and YW performed the statistical analysis and visualization of the data. XG wrote the manuscript. YY, LD, HZ, BX, YW, and JL revised the manuscript. The whole team approved the final version of the manuscript.
