# Supplementary material for: Sub-macroscopic skin presentation of acromegaly and effect of pituitary tumor surgery: A study using dermatoscopy and ultra-high-frequency ultrasound
Source: Front Endocrinol (Lausanne). 2023 Jan 10;13:1093942. doi: 10.3389/fendo.2022.1093942 (PMC9933496; doi:10.3389/fendo.2022.1093942)
Supplement: Supplementary file 1 [file Table_1.docx]

**Supplementary Table 1. Skin Lesions in Acromegaly Patients with and without Postoperative Endocrine Remission.**

|  | **Remission after Surgery (n=14)** | | |  | **Non-remission after Surgery (n=12)** | | | **P Value** |
| --- | --- | --- | --- | --- | --- | --- | --- | --- |
|  | **Baseline** | **Post-operation** | **P1 Value** |  | **Baseline** | **Post-operation** | **P2 Value** |  |
| **Megascopic Skin Presentation** |  |  |  |  |  |  |  |  |
| Thickened skin, n (%) | 14 (100) | 14 (100) | 1.000 |  | 12 (100) | 12 (100) | 1.000 | 1.000 |
| Face coarsening, n (%) | 14 (100) | 14 (100) | 1.000 |  | 11 (91.7) | 11 (91.7) | 1.000 | 0.462 |
| Enlarged hands and feet, n (%) | 14 (100) | 14 (100) | 1.000 |  | 12 (100) | 12 (100) | 1.000 | 1.000 |
| Excessive seborrhea, n (%) | 14 (100) | 12 (85.7) ↓ | 0.481 |  | 11 (91.7) | 8 (66.7) ↓ | 0.315 | 0.495 |
| Enlarged pores, n (%) | 13 (92.9) | 13 (92.9) | 1.000 |  | 10 (83.3) | 10 (83.3) | 1.000 | 0.887 |
| Hyperhidrosis, n (%) | 14 (100) | 11 (78.6) ↓ | 0.222 |  | 10 (83.3) | 7 (58.3) ↓ | 0.369 | 0.491 |
| Hypertrichosis, n (%) | 7 (50) | 7 (50) | 1.000 |  | 4 (33.3) | 4 (33.3) | 1.000 | 0.391 |
| Acne-like lesion, n (%) | 9 (64.3) | 6 (42.9) ↓ | 0.256 |  | 5 (41.7) | 1 (8.3) ↓ | 0.157 | 0.125 |
| Hyperpigmentation, n (%) | 10 (71.4) | 9 (64.3) ↓ | 1.000 |  | 8 (66.7) | 8 (66.7) | 1.000 | 1.000 |
| Facial erythema, n (%) | 12 (85.7) | 11 (78.6) ↓ | 1.000 |  | 9 (75) | 5 (41.7) ↓ | 0.098 | 0.128 |
| Acrochordon, n (%) | 2 (14.3) | 2 (14.3) | 1.000 |  | 2 (16.7) | 2 (16.7) | 1.000 | 1.000 |
| Keloid, n (%) | 3 (21.4) | 3 (21.4) | 1.000 |  | 2 (16.7) | 2 (16.7) | 1.000 | 1.000 |
| **Dermoscopic Skin Presentation** |  |  |  |  |  |  |  |  |
| Linear vessel, n (%) | 5 (35.7) | 4 (28.6) ↓ | 1.000 |  | 5 (41.7) | 4 (33.3) ↓ | 1.000 | 1.000 |
| Red structureless area, n (%) | 14 (100) | 13 (92.9) ↓ | 1.000 |  | 12 (100) | 11 (91.7) ↓ | 1.000 | 1.000 |
| Perifollicular orange halo, n (%) | 13 (92.9) | 12 (85.7) ↓ | 1.000 |  | 10 (83.3) | 7 (58.3) ↓ | 0.369 | 0.260 |
| Follicular plug, n (%) | 12 (85.7) | 11 (78.6) ↓ | 1.000 |  | 7 (58.3) | 6 (50) ↓ | 0.682 | 0.266 |
| Perifollicular pigmentation, n (%) | 13 (92.9) | 13 (92.9) | 1.000 |  | 11 (91.7) | 11 (91.7) | 1.000 | 1.000 |
| Increased hair shafts, n (%) | 8 (57.1) | 8 (57.1) | 1.000 |  | 6 (50) | 6 (50) | 1.000 | 0.716 |
| Honeycomb-like pigmentation, n (%) | 14 (100) | 14 (100) | 1.000 |  | 11 (91.7) | 11 (91.7) | 1.000 | 0.462 |
| Widened dermatoglyphics, n (%) | 11 (78.6) | 11 (78.6) | 1.000 |  | 10 (83.3) | 10 (83.3) | 1.000 | 1.000 |
| Dilated appendage opening, n (%) | 11 (78.6) | 11 (78.6) | 1.000 |  | 7 (58.3) | 6 (50) ↓ | 0.682 | 0.266 |
| **Ultrasonic Skin Presentation** |  |  |  |  |  |  |  |  |
| Skin thickness, mm | 4.0 ± 0.4 | 3.6 ± 0.4 ↓ | **0.045** |  | 4.1 ± 0.4 | 3.8 ± 0.4 ↓ | 0.080 | 0.383 |

↓ indicates the downward trend of the rate changes of skin presentations or skin thickness after surgery.

P1/P2 values indicate the differences of skin lesion rates and skin thickness between the pre- and the postoperative period in patients in remission (P1) and those not in remission (P2).

P value indicates the difference of postoperative rates of skin lesions and skin thickness between patients in remission and patients not in remission.
